# Sensitivity of occipito-temporal cortex, premotor and Broca’s areas to visible speech gestures in a familiar language

**DOI:** 10.1371/journal.pone.0234695

**Published:** 2020-06-19

**Authors:** Vincenzo Maffei, Iole Indovina, Elisabetta Mazzarella, Maria Assunta Giusti, Emiliano Macaluso, Francesco Lacquaniti, Paolo Viviani

**Affiliations:** 1 Laboratory of Neuromotor Physiology, IRCCS Santa Lucia Foundation, Rome, Italy; 2 Centre of Space BioMedicine and Department of Systems Medicine, University of Rome Tor Vergata, Rome, Italy; 3 Data Lake & BI, DOT - Technology, Poste Italiane, Rome, Italy; 4 Departmental Faculty of Medicine and Surgery, Saint Camillus International University of Health and Medical Sciences, Rome, Italy; 5 ImpAct Team, Lyon Neuroscience Research Center, Lyon, France; 6 Laboratory of Neuroimaging, IRCCS Santa Lucia Foundation, Rome, Italy; Universidad Complutense Madrid, SPAIN

## Abstract

When looking at a speaking person, the analysis of facial kinematics contributes to language discrimination and to the decoding of the time flow of visual speech. To disentangle these two factors, we investigated behavioural and fMRI responses to familiar and unfamiliar languages when observing speech gestures with natural or reversed kinematics. Twenty Italian volunteers viewed silent video-clips of speech shown as recorded (Forward, biological motion) or reversed in time (Backward, non-biological motion), in Italian (familiar language) or Arabic (non-familiar language). fMRI revealed that language (Italian/Arabic) and time-rendering (Forward/Backward) modulated distinct areas in the ventral occipito-temporal cortex, suggesting that visual speech analysis begins in this region, earlier than previously thought. Left premotor ventral (superior subdivision) and dorsal areas were preferentially activated with the familiar language independently of time-rendering, challenging the view that the role of these regions in speech processing is purely articulatory. The left premotor ventral region in the frontal operculum, thought to include part of the Broca’s area, responded to the natural familiar language, consistent with the hypothesis of motor simulation of speech gestures.

## Introduction

Watching the mouth movements of a speaker (so called, visual speech) may help listeners to decode speech in a noisy environment [1–3], and may even alter the auditory perception of speech as in the McGurk effect [4–9].

Observers can discriminate fairly reliably between silent video-clips of a speaker played as recorded (Forward mode) or time-reversed (Backward mode) [1]. It was argued that natural kinematics (recognition of biological motion) rather than linguistic competences had a role in this task [1].

Normal and time-reversed visual speech differ kinematically in several ways, although the qualitative differences are subtle. With few exceptions (such as long vowels or fricative consonants), phono-articulatory gestures tend to be asymmetric in time. For instance, deceleration phases are longer than acceleration phases [10], and asymmetries are present between the opening and closing movements of the mouth [11]. Moreover, articulatory gestures of speech obey specific constraints imposed by the motor system. Thus, the temporal inversion of these gestures often generates a sequence of unnatural movements hardly repeatable by normal people, although an experienced person can invert the temporal order of the phonemes in a sentence. In fact, the articulatory sequences that generate the phonemes during a speech are extremely complicated to perform in reverse. This is because one should reverse each phono-articulatory manoeuvre required to produce a given phoneme, as well as the specific sequence with which these manoeuvres are chained during the speech [1].

The central nervous system is also sensitive to language familiarity in visual speech. Indeed, a familiar language can be discriminated by the analysis of the speech temporal structure (i.e., rhythm) in auditory as well as in visual modality [12–14]. Temporal duration and variability of vowels and consonants differ between languages [15–20], and the timing of vowels and consonants can be visually assessed since phono-articulatory gestures generating these movements fit into different visual classes [2,12,21–24]. For instance, Spanish monolingual speakers visually distinguished Spanish from Catalan, while this was not possible either for English or for Italian speakers [12,25].

Discrimination ability of a familiar language persists also after a temporal reversal of visual speech stimuli [12]. The rhythmic and global timing structures of speech visible cues (e.g., alternation of consonants and vowels, vowels duration) remain relatively unaltered after a temporal inversion of speech sequences, while semantic, lexical and phonotactic information are lost [12]. Moreover, six-months-old infants are able to discriminate a familiar language from visual speech [26]. These observations suggest that visual spatio-temporal cues play a more important role in identifying familiarity than linguistic competence. The brain networks involved in these processes are unknown. To our knowledge, no study so far has directly investigated the neural correlates of language discrimination of visual speech, while the few papers reporting brain sites activated by inverting the temporal order of natural visual speech measured brain activity using techniques (PET or MEG) other than fMRI [27,28].

In theory, the occipito-temporal cortex (OTC) might have a specific selectivity to the spatio-temporal features of visual speech (i.e., kinematics of biological motion). Indeed, various foci in this region respond to different types of human movements and body forms [29–35]. Studies comparing face movements during a speech with facial movements that cannot be construed as speech reported activations in both lateral and ventral OTC, including the temporal visual speech area (TVSA) [36–38], as well as in auditory association areas of the temporal cortex in the superior temporal gyrus [37,39,40]. However, this comparison might be affected by the presence of confounds in low-level visual features, such as differences in motion speed [38,41]. A contrast immune from low-level visual confounds is the comparison of speech movements rendered normally (Forward) versus time-reversed (Backward).

A recent MEG experiment showed that, during the processing of silently played lip movements, the visual cortex tracks the missing acoustic speech information when played forward as compared to backward, indicating a top-down modulatory control of auditory dorsal stream on visual areas [42]. Also, in a PET study, the contrast Forward versus Backward engaged OTC bilaterally [27]. However, the ability to discriminate plausible speech gestures (i.e., Forward versus Backward video clips) was localised to later stages of processing, such as the parieto-temporal cortex and motor areas in the frontal cortex.

Visual speech stimuli have been shown to engage cortical motor areas involved in speech production, such as the left inferior frontal gyrus (IFG) that includes Brodmann’s Area BA 44 and BA 45 (pars opercularis and triangularis of IFG, respectively) thought to overlap with Broca’s region [43] and the ventral premotor cortex (PMv), but also more dorsal regions of the premotor cortex (PMd) [27,40,44–49]. Importantly, part of the frontal areas implicated in the control of movements and speech are connected with the visual cortex [50,51]. The motor theory of speech perception [52–54] proposed that the activation of motor speech areas during the observation of speech might represent an implicit motor simulation of the observed gestures conducive to speech understanding [44,55–57].

However, several authors questioned the idea that an automatic engagement of motor areas, such as IFG, during perceptual or cognitive task is evidence of a specific involvement of the motor system in perceptual or cognitive processes [28,58–64]. The dorsal premotor cortex, rather than the Broca’s area (BA 44/45), seems to be engaged both in the execution and the observation of speech gestures [62]. Conversely, it was found that the activity in the IFG correlates with hit-rate and response bias during speech perception tasks [27,65]. Since response bias and hit rate are characteristic indexes of the decisional process, these findings might suggest that high level processes related to the generation of the response decision (e.g. whether to respond Yes or No), rather than motor simulations occurred in the IFG during visual speech [66,67]. In summary, the specific role of IFG and Broca’s area in the functional architecture of speech perception remains open to debate [43,68].

In the present study, we investigated the neural circuits engaged by language familiarity (Italian vs Arabic) and natural kinematics of biological motion (Forward vs Backward) of visible speech. Italian observers viewed silent video-clips of the mouth movements of Italian and Arabic actors speaking in their native language. Stimuli were rendered either in normal (Forward) mode or after a time reversal (Backward). During an fMRI session, participants were asked to identify the rendering mode (Forward or Backward). The brain regions sensitive to language familiarity and those sensitive to natural mouth movements of speech were identified through the fMRI contrasts Italian vs Arabic (main effect of language) and Forward vs Backward (main effect of rendering mode), respectively. We also computed the interaction between the two main factors by means of the contrast “language x rendering mode” [(Italian Forward vs Italian Backward) vs (Arabic Forward vs Arabic Backward)]. The latter contrast should identify the areas where the effects of Familiarity (Italian vs Arabic) was larger for natural (Forward) than non-natural (Backward) motion (i.e biological motion).

## Methods

### Participants

Forty healthy right-handed Italian volunteers took part in this study. Twenty participants (14 females, 6 males; mean age: 25 years; age range: 20–42 years) were tested in a preliminary experiment and twenty different participants (13 females, 7 males; mean age: 23 years; age range: 20–35) in the main study (i.e. fMRI and in a follow-up experiment, see later). All participants had normal or corrected to normal vision. None of them had any familiarity with the Arabic language or experience with lip-reading. Written informed consent to procedures approved by the Institutional Review Board of Fondazione Santa Lucia was obtained from each participant. Experimental protocols complied with the Declaration of Helsinki on the use of human subjects in research.

### Stimuli

Ten adults (5 females, 5 males) native speakers of Arabic and 10 adults (5 females, 5 males) native speakers of Italian volunteered as actors for generating the stimuli. We chose the Arabic rather than other European languages in order to ensure that the participants would have not been exposed more than occasionally to this language before, and we verified that this was the case. The choice of the Arabic was also motivated because it differed from the Italian more than did most other European languages. Indeed, articulatory movements for the production of words in Arabic and Italian languages are different [18,20].

Two of the authors (P.V. and V.M.) selected Italian speakers so that, after careful visual inspection, the general features of the lower part of the face were roughly similar to those of the Arabic speakers previously selected. None of the actors participated in the main or in the preliminary study. Each actor read four texts excerpted from newspapers in his/her native language (Arabic, A or Italian, I). The preparation of the stimuli involved three steps. First, we recorded the lower part of the face (including the upper/lower lips and the chin) of each actor with a digital camera (25 frames/s, Sony HDR-SR-8E), and stored the results as a sequence of single frames (1024 x 768 pixel, RGB TIFF format). Second, static frames were processed with Photoshop CS6 to equalize for luminance and chromatic spectrum, and cropped to the size of 972 x 694 pixels in order to display only the mouth movements. Finally, by using Virtual Dub, we transformed the sequences of frames into silent AVI video-clips lasting 14 s. The experimental stimuli consisted in the video-clips rendered either as recorded (Forward mode, F) or after reversing the frames order (Backward mode, B). The total number of available stimuli was 2 [rendering mode] x 2 [language] x 10 [actor] x 4 [text] = 160. Four examples of video stimuli, one for each category of interest (I_F_, I_B_, A_F_, A_B_), are provided as supplementary material.

Additionally, we checked whether Arabic and Italian video-clips differed in motion energy. For each two consecutive frames of each video-clip, we calculated the mean of the squared differences in the red, green, and blue channels in every pixel [69–72]. The motion energy was estimated as the average of these values across all pixels and frames (350 frames) of each video. We found that the motion energy was not significantly different (unpaired t-test; p = 0.7; t-value (78) = 0.38) between Italian (mean ± SD: 34.0 ± 11.8 pixel^2^/frame^2^) and Arabic videos (34.9 ± 7.8 pixel^2^/frame^2^).

### General outline of the study

The study involved three successive experiments: a preliminary, purely behavioural session with the first group of 20 participants in which we estimated the ability to discriminate presentation modes (Backward/Forward); a main session with the second group of 20 participants in which this ability was estimated while measuring brain activity with fMRI; a follow-up session with the same participants of the fMRI session in which we estimated the ability to discriminate language familiarity (Italian/Arabic).

### Main task: Identification of rendering mode

In both fMRI and preliminary experiments, participants were informed that the video-clips being shown could be either a faithful or a time-reversed rendering of actual speech movements. They were not informed that in half of the videos the actor’s language was Italian (I) and in remaining half was Arabic (A). The task (2-Alternative Forced Choice: 2-AFC) was to indicate whether the video was displayed as recorded (Forward) or reversed in time (Backward). Participants had to wait until the end of the stimulus before responding. Responses were entered by pressing with the right index finger one of two buttons marked “F” (Forward) and “B” (Backward), respectively. Between trials, the display was uniformly grey and participants fixated a central point (0.5° visual angle). No constraints were imposed on oculomotor behaviour during the presentation of the stimuli. Before each experimental session, participants were administered eight warm-up trials, which included at least one example for each combination of actor gender, native language, and rendering mode. The results of these trials were not analysed.

### Follow-up task: Identification of language

The participants in the fMRI experiment were retested 10 (± 2) days later in a follow-up experiment outside the scanner. They viewed the same 160 silent video-clips described above. Participants were informed that the actor’s language could be either Italian or another, unspecified language, but no further information was provided. Participants were asked to wait until the end of the stimulus before responding (2-AFC). Reponses were entered by pressing with the right index finger a button marked either I (Italian) or NI (not Italian). The aim of this experiment was to gauge the accuracy with which viewers could discriminate a familiar language (Italian) from an unfamiliar one (Arabic) using only visual cues, and to test whether the time-arrow (forward vs. backward rendering mode) affects the judgment of language familiarity. We hypothesized that the visual cues used to discriminate languages (e.g., temporal variability of vowels) are different from those used for discrimination of rendering mode (e.g., the acceleration profiles of opening/closing movements of the mouth). If so, the sensitivity index (see below) should be uncorrelated between the two tasks.

### General procedure

In each experiment, the total number of stimuli (160) was divided in 5 runs (32 stimuli/run) with the constraint that successive stimuli never involved the same actor. Stimuli were pseudorandomized and presented using the Presentation software (Neurobehavioural system^®^). Within runs, interstimulus intervals (ISI) followed a uniform distribution (range: 2 s–4 s; mean: 3 s). The five runs were administered in a single session and were separated by brief pauses. Additionally, in the fMRI experiment, to estimate more accurately the shape of BOLD impulse response [73–75], we pseudo-randomly inter-mixed null events (N = 35, duration 8 s). Thus, the duration of each run was 10’ 50” during fMRI and 9’ 54” in the follow-up and preliminary experiments.

### fMRI experiment: Set-up

Participants lay supine in the MR scanner with the head immobilized with foam cushioning and wore earplugs and headphones to suppress ambient noise. A digital projector (NEC LT158, refresh rate: 60-Hz) projected the stimuli through an inverted telephoto lens onto a semi-opaque Plexiglas screen mounted vertically inside the scanner bore, behind the participant’s head. The back-projected image was then viewed via a mirror mounted on the head coil positioned at about 4.5 cm from the eyes. The eye-to-screen equivalent distance was 66 cm, and the angular size of the projected image was 9° (width) × 6.4° (height). Responses were acquired with an MR-compatible response box (fORP, Current Designs).

### Follow-up experiment and preliminary experiment: Set-up

The follow-up and preliminary experiments were performed in a quiet, dimly illuminated room. Participants sat in front a 19” LCD monitor and viewed the silent video-clips (9° x 6.4° visual angle) in a pseudo-random order at a distance of about 80 cm. Responses were entered via a high-speed button box (Empirisoft^®^).

### Behavioural data analysis

In the fMRI experiment and in the preliminary experiments, where the task was to identify the rendering mode (see above), responses were collated by language, rendering mode and actor. The number of responses “Forward” to Forward and Backward stimuli are indicated as N_F|F_ and N_F|B_, respectively, while the number of responses “Backward” to Forward and Backward stimuli for each language are indicated as N_B|F_ and N_B|B_, respectively. For each participant, the sample size was N_T_ = 80 for each language, thus a total of 1600 trials for each language was collected (N_T_ x 20 participants). For each participant, we computed a sensitivity index d’ = Z{Hit} − Z{False Alarm} and a response bias *c* = −0.5*(Z{Hits} + Z{False Alarm}), where Z{Hit} and Z{False Alarm} are the z-scored transformed values of P{Hit} = P{F|F} = N_F|F_/N_T_, and P{False Alarm} = P{F|B} = N_F|B_/N_T_, respectively [76]. Moreover, we calculated the probability of correct responses as P{C} = (N_F|F_ + N_B|B_) / N_T_.

Similarly, in the follow-up experiment where the task was to identify the actor’s language (see above), responses were collated by language, rendering mode and actor. The number of responses “Italian” to Italian and Arabic stimuli are denoted as N_I|I_ and N_I|A_, respectively, and N_A|I_ and N_A|A_ are the number of responses “Not Italian” to Italian and Arabic stimuli, respectively. For each participant, the sample size was N_T_ = 80 for each rendering mode, thus and a total of 1600 trials for each rendering mode was collected (N_T_ x 20 participants). We estimated sensitivity and response bias through d’ and *c* indexes, respectively, based on the convention that, in this case, Z{Hit} and Z{False Alarm} are the z-scored transformed values of P{Hit} = P{I|I} = N_I|I_ / N_T_ and P{False Alarm} = P{I|A} = N_I|A_ / N_T_, respectively. Moreover, we calculated the probability of correct responses: P{C} = (N_I|I_ + N_A|A_) / N_T_.

We considered d’ and response bias in addition to the probability of correct responses, since the latter might be inflated by response bias and lead to misleading interpretations [76].

We expected that responses to the stimuli depended on whether participants had to discriminate between rendering mode (in the first two experiments) or languages (in the follow-up experiment). Thus, within-subject responses to the rendering-mode and language discrimination task should show different patterns, and the sensitivity index (d’) should be uncorrelated between tasks. To verify these points, we calculated the correlation coefficient of participants’ sensitivity index between the main task and the follow-up experiment task.

### fMRI data acquisitions

MR images were acquired with a Siemens Magnetom Allegra 3T head-only scanning system (Siemens Medical Systems, Erlangen, Germany), equipped with a quadrature volume RF head coil. Whole brain BOLD echoplanar imaging (EPI) functional data were acquired with a 3T-optimized gradient echo pulse-sequence (TR = 2.47 s, TE = 30 ms; flip angle = 70°; FOV = 192mm, fat suppression). 38 slices of BOLD images (volumes) were acquired in ascending order (64 x 64 voxels, 3 x 3 x 2.5 mm^3^, distance factor: 50%; inter-slice gap = 1.25 mm; slice thickness = 2.5 mm), covering the whole brain. For each participant, a total of 1315 volumes of functional data were acquired in five consecutive runs. At the end of each run, the acquisition was paused briefly. Structural MRI data were acquired using a standard T1-weighted scanning sequence of 1 mm^3^ resolution (MPRAGE; TE = 2.74 ms, TR = 2500 ms, inversion time = 900 ms; flip angle = 8°; FOV = 256 × 208 × 176 mm^3^).

### fMRI data preprocessing

Data and statistical analyses were performed using the SPM12 software (Wellcome Trust Centre for Neuroimaging, London, UK) implemented in MATLAB R2013 (The MathWorks Inc., Natick, MA) using standard procedures [77,78]. After discarding the first four volumes of each run, images were corrected for head movements, realigned to the mean image, coregistered to the structural image, and normalized to Montreal Neurological Institute (MNI) space using unified segmentation [79], including resampling to 2 × 2 × 2 mm voxels, and spatially smoothed with a 8 mm full-width at half maximum (FWHM) isotropic Gaussian kernel. Voxel time series were processed to remove autocorrelation using a first-order autoregressive model and high-pass filtered (128-s cut-off).

### fMRI analysis

Patterns of brain activations were computed using the general linear model and a Finite Impulse Response (FIR) set of base functions. Here, the FIR approach is ideal to fit brain activity, because it can identify changes of activity over time without making any assumptions about the profile of these changes [80]. Accordingly, for each participant, the FIR estimated the level of activation in 12 successive time-bins. Each time-bin consisted of 1 TR (2.47 s), thus fitting 30 s of the fMRI data for each stimulus. We modelled 5 different event-types: Italian Forward rendering (I_F_), Italian Backward rendering (I_B_), Arabic Forward rendering (A_F_), Arabic Backward rendering (A_B_), time-locked to stimulus onset, thus obtaining 12 images (one for each time bin) for each correct trial of the 4 conditions, plus an additional event corresponding to errors trials irrespective of condition. Motion correction parameters were also included as effects of no interest. We analysed the activity related only to stimuli correctly identified (correct trials), since error trials (stimuli not correctly identified) may introduce confounding activation (i.e. contamination of the activation related to poorer performance by increased errors [81,82]. However, to evaluate the effect of error trials on the fMRI activity, we did a supplementary analysis (not reported here) with all trials (correct and error trials). We found that the brain sites activated in the main fMRI analysis (see Results and Table 1) were also activated in the supplementary fMRI analysis, although at an uncorrected level (p-uncorr < 0.05), thus indicating that error trials decrease the signal-to-noise ratio.

**Table 1:** Peaks of cluster activations.

**Actor’s language (Italian Vs Arabic)**						
*Anatomical Area*	*x*	*y*	*z*	*k*	*F-value*	*FWE corr*
FGa	-34	-66	-14	265	6.44	Whole brain
IOG	-34	-80	-14		3.50	
OTS	38	-72	-4	353	5.33	Whole brain
FGa	38	-74	-12		4.95	
IOG	40	-80	10		3.70	
Precuneus	0	-64	44	169	4.06	Whole brain
	-8	-60	44		3.67	
	-4	-52	42		3.44	
PMvs/PMd	-44	6	50	84	4.54	ROIs
**Rendering mode (Forward vs Backward)**						
	*x*	*y*	*z*	*k*	*F-value*	*FWE corr*
IPS	-24	-66	60	376	6.16	Whole brain
	-28	-64	38		3.99	
	-18	-60	46		3.42	
FGb	-30	-60	-4	137	4.98	Whole brain
IPS	30	-68	42	224	4.35	
	32	-70	34		4.08	
	30	-58	44		3.26	
Pmvi	-56	10	6	111	4.09	ROIs
**Interaction: Actor’s language x Rendering mode**						
	*x*	*y*	*z*	*k*	*F-value*	*FWE corr*
IFG	-40	38	10	193	6.12	Whole brain
	-48	34	4		3.73	
LG	-18	-62	0	464	5.46	Whole brain
	-26	-66	8		3.91	
	-22	-52	-6		3.29	

FG = Fusiform Gyrus, OTS = Occipito—Temporal Sulcus, IPS = Intra-Parietal Sulcus, LG = Lingual gyrus, IFG = Inferior Frontal gyrus; *k = cluster size (in voxels)*. *Family wise error correction (FWE) at p < 0*.*05 can be whole brain or within ROIs*.

At single-subject level, we estimated four effects of interest. First, we calculated the contrast representing the overall mean activity of all stimuli by averaging the estimated parameter of all conditions ([I_F_ + I_B_ + A_F_ + A_B_]/4) in each bin. Subsequently, we estimated the contrasts of the three effects: (1) main effect of actor’s language ([I_F_ + I_B_] vs. [A_F_ + A_B_]); (2) main effect of rendering mode ([I_F_ + A_F_] vs. [I_B_ + A_B_]); and (3) modulatory effect of actor’s language on rendering mode (interaction: [I_F_ − I_B_] vs. [A_F_ − A_B_]). The resulting parameters for each contrast (corresponding to 12 images, one for each time bin) in each participant were then entered into second-level group analyses [83].

Four separate one-way ANOVAs with 12 levels (each corresponding to one time-bin) were performed at the second (group) level. We used F-contrasts to highlight brain areas showing differential activity over the 12 time-bins, separately for each of the four ANOVAs. In particular F-contrasts subtracted the activity of the first bin (i.e. one TR at stimulus onset) from each of the other bins, thus capturing the changes of activity over-time. All analyses included appropriate corrections for non-sphericity. Statistical thresholds were set at p-FWE < 0.05, family-wise error corrected for multiple comparisons at cluster level (hereafter, p-corr < 0.05), using a voxel-wise threshold set at p< 0.001 [84,85]. Furthermore, post-hoc t-tests on each time bin were false-discovery-rate (FDR) corrected for n multiple comparisons at p < 0.05 across the number of bins (n = 12).

### Regions of interest

In addition to the previous whole-brain analysis, we also performed an analysis based on regions of interest (ROIs). In particular, we defined regions as spheres of 8 mm radius centred on premotor areas that respond to visual speech (Premotor Ventral inferior PMvi/Broca’s xyz = −48 12 9, xyz = −51 9 9; Premotor ventral superior / premotor dorsal PMvs/PMd xyz = −39 3 54, xyz = −48 3 42; BA6 and BA 44 xyz = 48 18 18) [86]; visual motion area MT+/V5 (xyz = -42–66 2, xyz = 42–62 6) [87], and sites in the posterior inferior temporal sulcus involved in biological motion processing (pITS xyz = -50–82 0, xyz = 48–78–4) [88]. Finally, we considered ROIs also in the fusiform face area (FFA xyz = -34–62–15, xyz = 34–62–15) [89] and in the temporal visual speech area (TVSA xyz = -57–34 14) [90]. We applied family-wise-error small-volume-correction (FWE-SVC) to each ROI [91,92]. We retained results as significant at p < 0.05 FWE-SVC, further Bonferroni-corrected for the number of regions (n = 12).

## Results

### Behavioural results

#### Main task: Identification of the rendering mode (Forward or Backward)

During the preliminary and fMRI experiments, observers had to indicate whether the video-clip was played in Forward or Backward mode.

**Fig 1:**
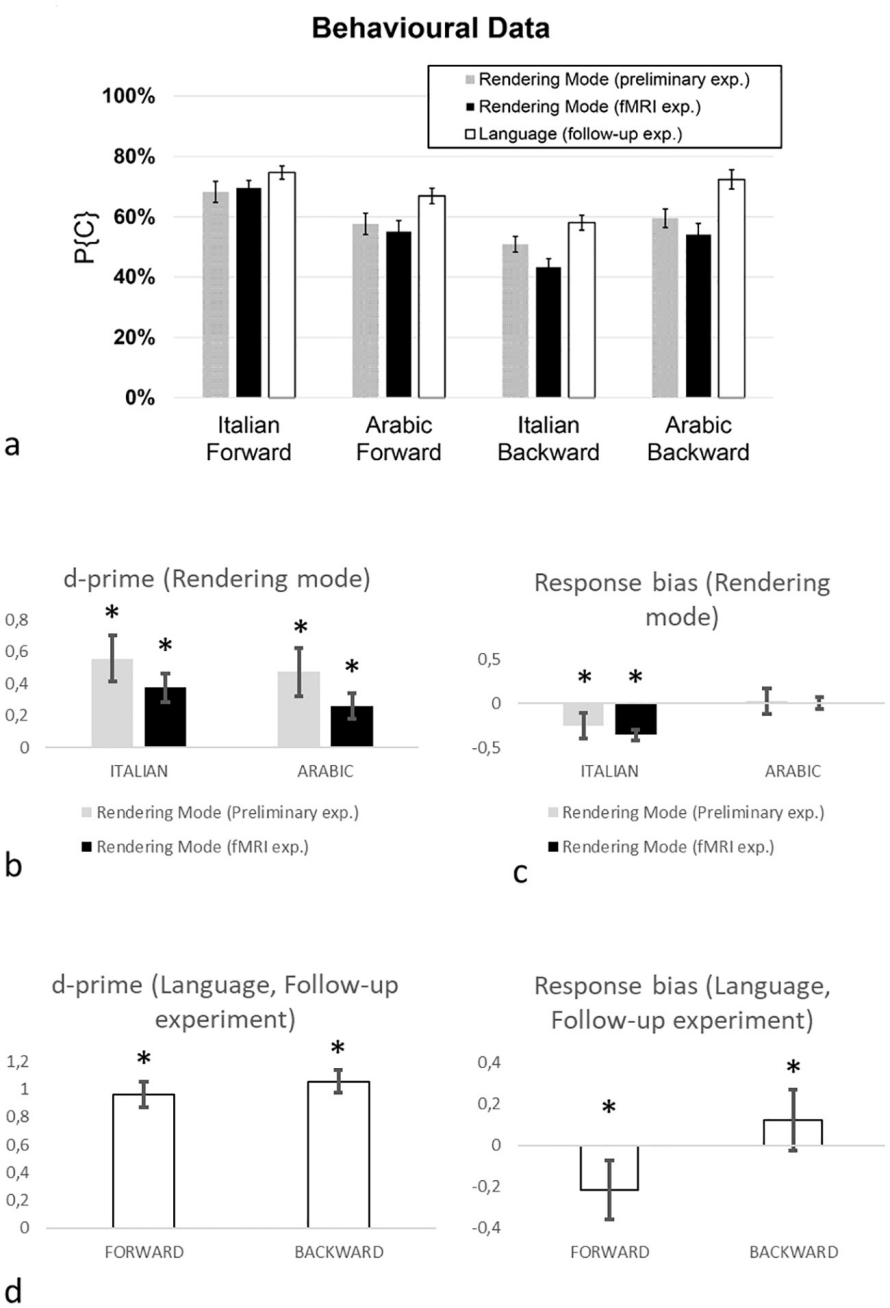
(a) Proportion of correct responses (mean ± s.e.m.) separate for conditions and experiment. (b) D-prime sensitivity to rendering modality in the preliminary and fMRI experiment. (c) Response bias to language during the sensory modality in the preliminary and fMRI experiment. (d) D-prime sensitivity to language and response bias to rendering modality during the follow-up experiment. Asterisks indicate significant values.

Observers detected the rendering mode (Forward or Backward) of video-clips with an overall probability of correct responses P{C} = 0.590 and P{C} = 0.556 for the preliminary and fMRI experiments respectively (pooled across participants and stimuli), significantly higher than chance level (two-tailed binomial test, p < 0.001, Fig 1a). Sensitivity (d’) for Italian (d’: 0.56 ± 0.14 and d’: 0.37 ± 0.09, mean ± s.e.m., for preliminary and fMRI experiments respectively) and Arabic (d’: 0.47 ± 0.15 and d’: 0.26 ± 0.08, respectively) was not significantly different (paired t-test; p = 0.52; t(19) = 0.66 and p = 0.16; t(19) = 1.44 for preliminary and fMRI experiments respectively, Fig 1b). For both languages, d’ was significantly greater than 0 (one sample t-test; all p <0.002; t(19) > 3.25 and all p <0.004; t(19) > 3.32 for preliminary and fMRI experiments respectively). However, there was a significant response bias (*c* = -0.25 ± 0.07, p = 0.003, t(19) = 3.34 and *c* = -0.36 ± 0.05, p = 0.001, t(19) = 4.05 one-sample t-test, for preliminary and fMRI experiments respectively) in favour of the response “Forward” for the Italian video-clips (Fig 1c), underlying the higher proportion of correct response in this task. By contrast, there was no response bias for the Arabic video-clips (*c* = 0.024 ± 0.06, p = 0.66, t(19) = 0.43 and *c*: -0.01 ± 0.08, p = 0.9, t(19) = 0.12, one-sample t-test, for preliminary and fMRI experiments respectively).

The comparison of sensitivity (d’) and response bias (c) indexes between the fMRI and the preliminary experiments, computed for both Italian and Arabic video clips, did not show significant differences (t-test, all t(19) < 1.28, p>0.21).

#### Follow-up experiment: Language identification

All the participants in the fMRI experiment were retested in a follow-up experiment to ascertain their ability to recognize the language by means of visual-only cues. In this experiment, volunteers had to indicate if the actor’s language in the silent video clips was Italian or not. Fig 1a (white bars) reports for each condition the probability of correct responses (P{C} = 0.679) pooled across stimuli and participants. In all conditions, P{C} was significantly higher than chance level (two-tailed binomial test, p < 0.001). The average d’ was not significantly different between video-clips played in forward (d’: 1.05 ± 0.12) and backward mode (d’: 0.96 ± 0.15) (paired t-test; p = 0.5; t(19) = 0.65) and in both cases d’ > 0 (one sample t-test; all p <0.001; t(19) >7.02) (Fig 1d). There was a significant response bias in favour of the response “Italian” in the case of Forward video-clip (c: -0.21 ± 0.05, p < 0.001, t(19) = 4.43, one-sample t-test). Conversely, in the case of Backward video-clips, the response bias was in favour of the response “not Italian” (c: 0.12 ± 0.034, p < 0.01, t(19) = 2.95, one-sample t-test).

#### Comparison between tasks

We expected that stimuli were classified differently depending on the task, and that the two tasks had different response patterns across subjects. To verify this hypothesis, we compared the participants’ sensitivity index (d’) in the fMRI main task (rendering mode discrimination) and in the follow-up experiment (language discrimination). The analysis of d’ showed a greater sensitivity to stimuli in the follow-up experiment task compared to stimuli sensitivity in the main task (paired t-test, t(19) = 6.15, p<0.001). An alternative possibility is that the higher d’ in the second experiment could be due to a learning process occurring after the first experiment. In this case, we should expect a correlation across participants between tasks. However, the sensitivity indexes of the two tasks were not correlated (Pearson’s r = 0.27, p = 0.23), suggesting that the two tasks rely on different processing.

### fMRI results

#### Brain areas engaged by visual speech

**Fig 2:**
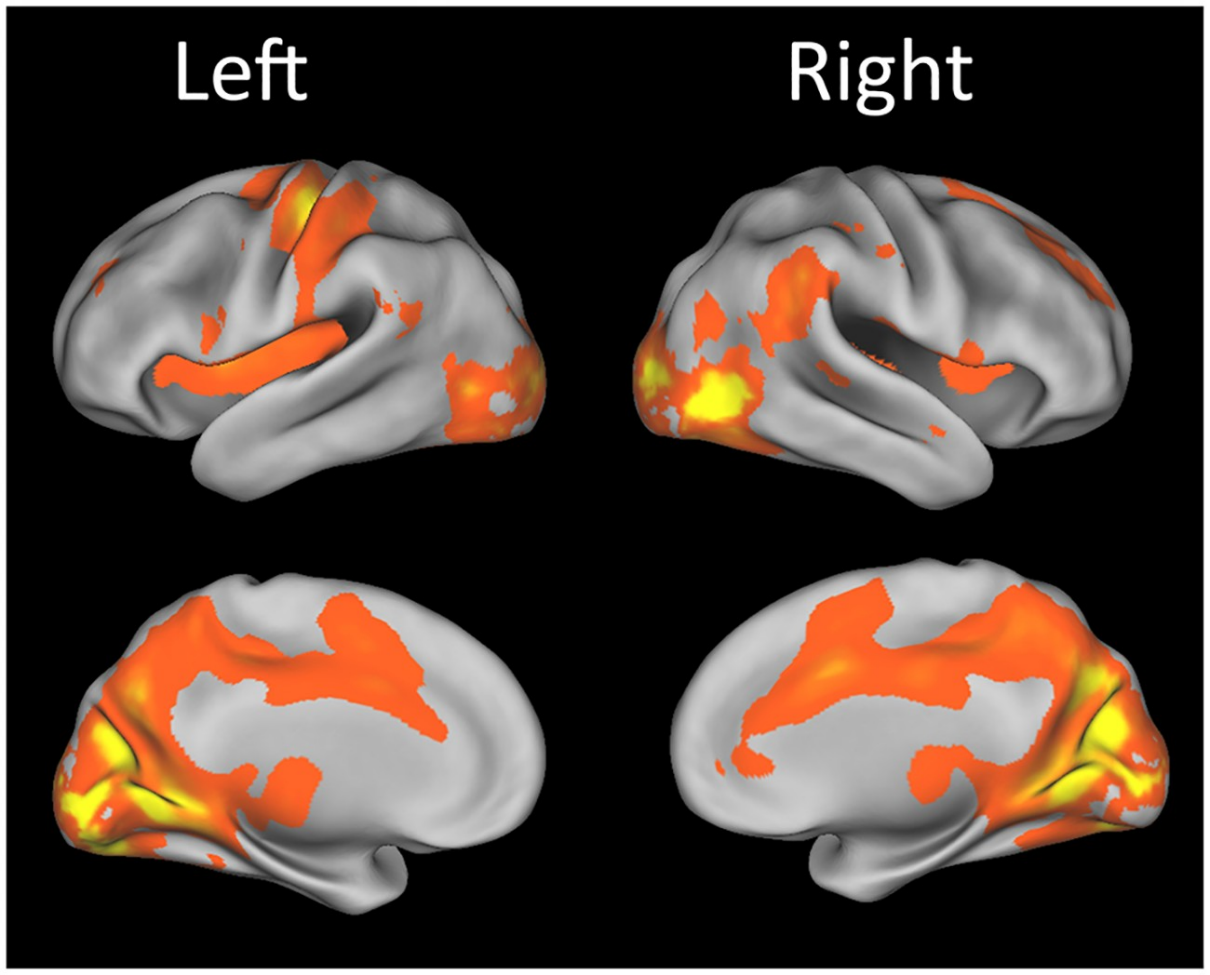
Statistical parametric mapping of GLM analysis. Effects of all stimuli vs. rest (F-test, p-corr < 0.05). Activations maps were overlaid on the standardized (inflated) brain of the PALS-B12 atlas implemented in Caret5 (Van Essen et al. 2005).

We mapped the cortical regions activated by all visual speech stimuli, irrespective of the parameters manipulated experimentally, (i.e., all stimuli vs. rest) by a differential F-test across the 12 time-bins (see Methods). This test highlights regions having different amplitude and/or time-course of the BOLD response between conditions examined in the contrast image (in this case, all stimuli vs. rest condition). As shown in Fig 2, significant effects were observed in occipital and temporal cortices, i.e. regions that are typically involved in audio-visual processing, as well as in parieto-frontal cortices, which are generally engaged by the vision of speech movements (Bernstein et al 2014), in the insula, cingulate cortex, motor and premotor areas. Activity in left motor/premotor areas was presumably related, at least in part, to the right-hand motor responses.

#### Main effect of actor’s language

**Fig 3:**
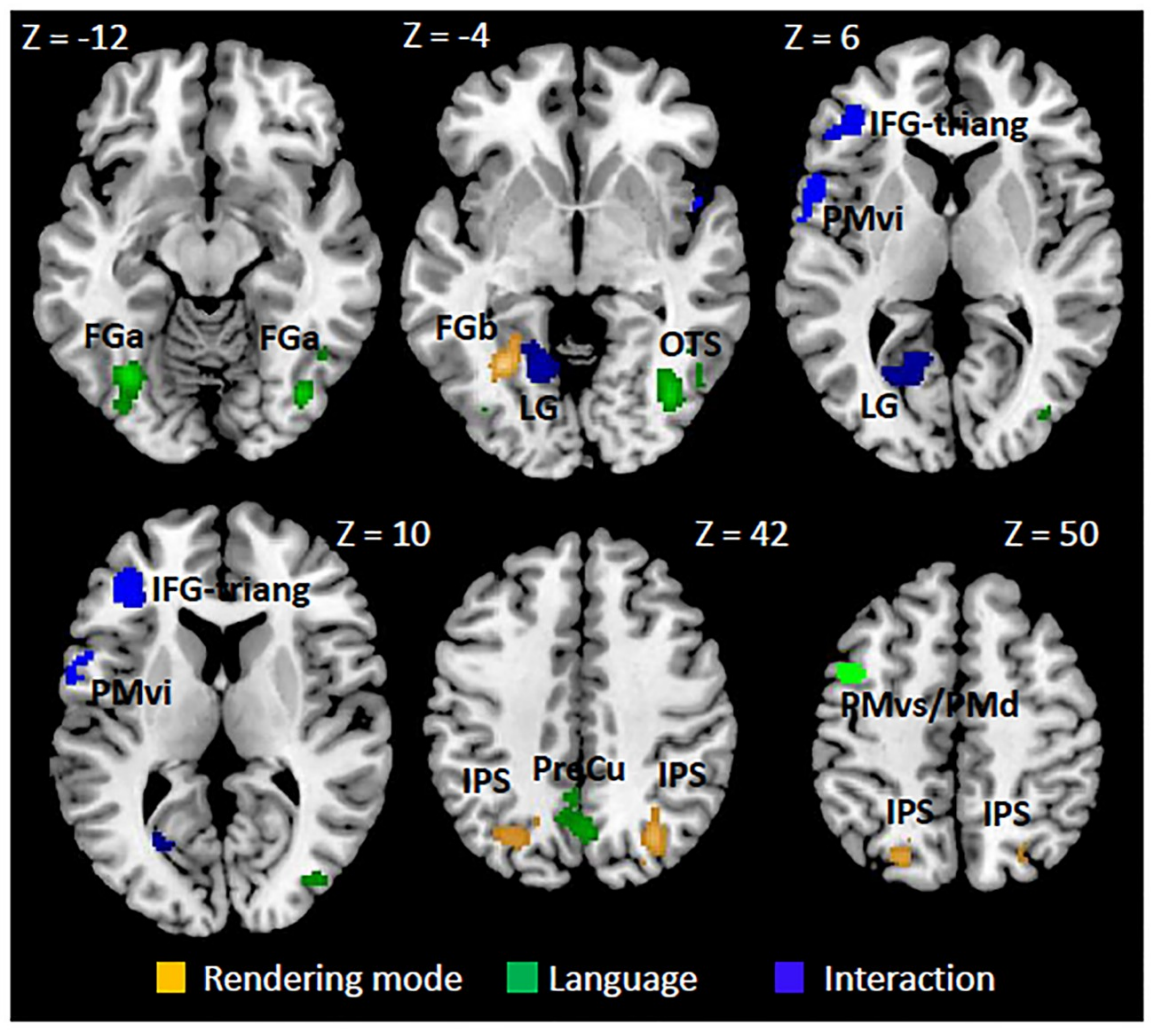
Statistical parametric mapping of GLM analysis. Main effects of actor’ language (green), rendering mode (orange) and the interaction actor’s language x rendering mode (blue), (F-test, p-corr < 0.05). Maps are projected onto the coronal slices of the 152-MNI template. Z-coordinate of each slice is reported on the top (in mm). FG_a,b_: fusiform gyrus; IFG-triang: inferior frontal gyrus pars triangularis; OTS: Occipito-Temporal Sulcus; PreCu: precuneus; IPS: intraparietal sulcus; LG: lingual gyrus; PMvi: premotor ventral inferior; PMvs/PMd: premotor ventral superior/Premotor dorsal.

**Fig 4:**
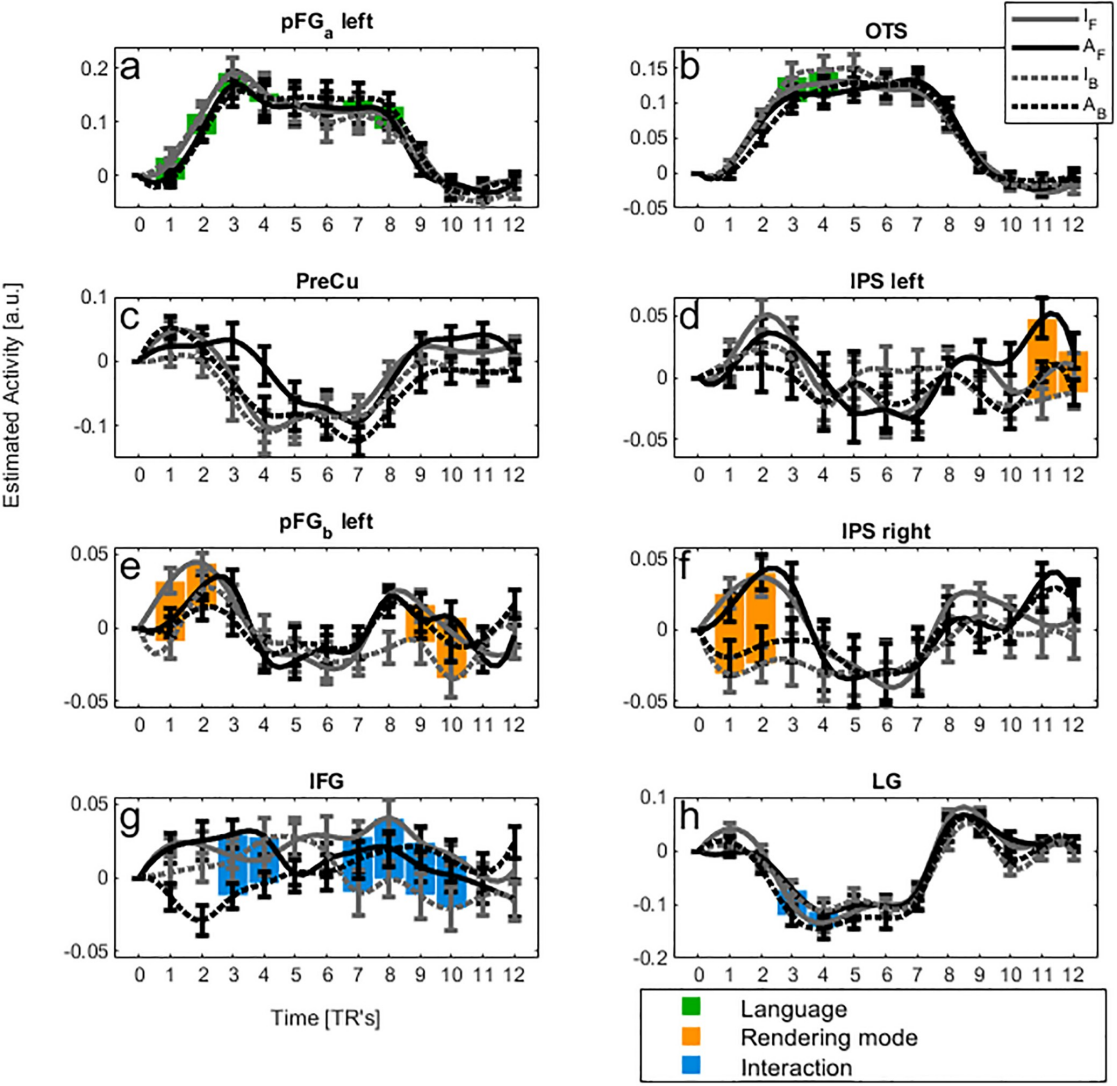
Peri-Stimulus Time Histogram (PSTH) for regions that showed significant effects in the whole-brain analysis. Mean time course (± s.e.m) of estimated BOLD signal at the peak voxel (see Table 1). Abscissa: time in TR’s unit (TR = 2.47s), T = 0: trial onset. Continuous and dotted lines indicate forward and backward rendering mode, respectively. Black and grey lines indicate Arabic and Italian video-clips, respectively. Green, orange and blue filled rectangles indicate time bins showing a significant difference in BOLD activity (post-hoc t-test, p-value FDR-corrected for multiple comparison < 0.05) due to Actor’s language, rendering mode or interaction, respectively. I_F_: Italian forward, A_F_: Arabic forward, I_B_: Italian Backward, A_B_: Arabic Backward.

*Whole brain analysis*. The differential F-test comparing Italian (familiar) vs. Arabic (unfamiliar) stimuli (irrespective of rendering mode) across the 12 time-bins revealed significant activations (p-corr < 0.05, whole brain) bilaterally in the posterior fusiform gyrus (FG_a_, at coordinates almost coinciding with those of FFA), extending to the inferior occipital gyrus (IOG) and right occipito-temporal sulcus (OTS, at coordinates near to those of pITS, a biological motion sensitive area), and in the precuneus (Fig 3, green area, Table 1). The time profiles of the estimated BOLD activity in these regions are plotted in Fig 4a, 4b and 4c. It is important to note that direct inspection of these activity patterns is necessary before any conclusion can be drawn, since significant differences obtained through our statistical analysis (i.e., F-test) could be due to a modulation in amplitude and/or to a time-shift. Time bins presenting a different activity level (post-hoc t-test, p-FDR corrected for multiple comparison < 0.05 across bins) between Italian and Arabic are filled in green. FG_a_ showed enhanced earlier activity (see bins 1^th^–3^th^) for Italian stimuli independently of rendering mode, and later activity for Arabic stimuli (6^th^–8^th^ bins, compare grey and black lines in Fig 4). IOG and OTS, belonging to the same cluster of FG_a_, had a similar temporal profile (e.g., OTS in Fig 4). The precuneus showed the opposite trend, with a decreased activity in the earlier bins for Italian stimuli (see black lines in Fig 4c, 3^th^–4^th^ bins) and in the later bins for Arabic stimuli (7^th^–8^th^ bins).

**Fig 5:**
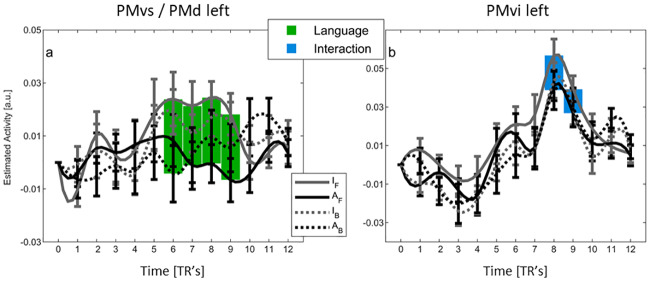
Peri-Stimulus Time Histogram (PSTH) for regions that showed significant effects in the ROI analysis. Mean time course (± s.e.m) of estimated BOLD signal at the peak voxel (see Table 1). Abscissa: time in TR’s unit (TR = 2.47s), T = 0: trial onset. Continuous and dotted lines indicate forward and backward rendering mode, respectively. Black and grey lines indicate Arabic and Italian video-clips, respectively. Green and blue filled rectangles indicate time bins showing a significant difference in BOLD activity (post-hoc t-test, p-value FDR-corrected for multiple comparison < 0.05) due to Actor’s language or interaction, respectively. I_F_: Italian forward, A_F_: Arabic forward, I_B_: Italian Backward, A_B_: Arabic Backward.

*ROI analysis*. A significant effect of the familiar language independently of rendering mode (p-corr < 0.05, FWE-SVC Bonferroni) was also found in the left PMvs/PMd (Table 1, Fig 3, green area). These regions showed increased activity for the Italian stimuli independently of rendering mode only in late bins (6^th^–9^th^ bins) (Fig 5a).

Also left FFA and right pITS showed a main effect of language, already reported in the whole brain analysis. By contrast PMvi, MT+/V5 and TVSA did not show a significant main effect of language.

#### Main effect of rendering mode

*Whole brain analysis*. Regions with differential responses to normal kinematics (i.e., video-clips played forward) and to implausible kinematics (i.e., video-clips played backward) were identified by the contrast Forward vs. Backward mode (main effect of rendering mode), irrespective of language (Italian or Arabic). The regions significantly sensitive to this contrast (orange regions in Fig 3) were found in the intraparietal sulcus (IPS) bilaterally, and in the left posterior-middle fusiform gyrus (FG_b_) (p-corr < 0.05, whole brain). Fig 4d, 4e and 4f show the time-course of activity in these regions. Bins in which the activity differed significantly between normal and reversed video-clips are filled in orange (post-hoc t-test, p<0.05 FDR corrected for multiple comparisons across bins). In particular, the right IPS (1^th^–2^th^ bins) and FG_b_ (1^th^–2^th^ bins) responded more to Forward than Backward rendering mode (compare continuous and dotted lines in Fig 4e and 4f, respectively). Left IPS showed a similar trend in the early bins (2^th^–3^th^ bins) at a lower statistical threshold (p-uncorr < 0.05).

Finally, the image resulting from the intersections (logical AND) between the cluster image of the left FG_a_ (reported above) and the cluster image of left FG_b_ showed that these two clusters were sharply separated (no voxel in common).

*ROI analysis*. A significant effect of the rendering mode independently of language (p-corr < 0.05, FWE-SVC Bonferroni) was also found in the PMvi, in the pars opercularis of IFG (p-corr < 0.05, FWE-SVC Bonferroni) (see Table 1, Figs 3 and 5b, blue). PMvi responded more during late bins to the rendering modality (8^th^–9^th^ bins), but selectively to Italian language, so that also the interaction calculated on the peak was significant (see also below).

By contrast none of the posterior ROIs (MT+/V5, FFA, pITS, TVSA) nor PMvs/PMd showed a main effect of rendering.

#### Influence of actor’s language on the rendering mode discrimination process

*Whole brain analysis*. Through the contrast ([I_F_ − I_B_] vs. [A_F_ − A_B_]), we searched for brain sites where the response to rendering mode was affected by language. This analysis revealed significant activations (p-corr < 0.05) in pars triangularis (BA 45) of the left inferior frontal gyrus (IFG-triang) and in lingual gyrus (LG, see blue regions in Fig 3). The temporal profile of BOLD responses (Fig 4g) showed that IFG-triang differentiated the Arabic video-clips played in Backward and Forward mode in the earlier bins, in particular Arabic backward stimuli strongly de-activated IFG-triang (3^th^–4^th^ bins) (post-hoc t-test, p-FDR corrected for multiple comparison < 0.05 across bins). In a similar way, IFG-triang differentiated Forward from Backward Italian video-clips, but at later times compared to Arabic stimuli discrimination, namely between the 8^th^ and 10^th^ bins (Fig 4g). Moreover, Italian stimuli played backward also showed a marked negative pattern in these bins. Overall, IFG-triang responded similarly to Italian and Arabic stimuli, although the temporal patterns were shifted. Indeed, neither the difference between the maximum peaks for Italian Forward stimuli (bin 8) and Arabic Forward stimuli (bin 3) (t(19) = 0.93; p = 0.36), nor the difference between Forward and Backward condition of Italian stimuli at bin 8 and that of Arabic stimuli at bin 3 (t(19) 0.12; p > 0.9) were significantly different (compare the differences between continuous and dotted grey lines in bin 8 and between continuous and dotted black lines in bin 3, respectively). In sum, the BOLD patterns showed that IFG-triang does not have a clear preferential response to the speech gestures most frequently performed by participants (i.e., Italian Forward stimuli).

LG showed a general de-activation in all four conditions versus rest. In particular, Italian Backward and Arabic Forward stimuli involved a very similar time-course, as did Arabic video-clips played backwards and Italian video-clips played forwards. These two latter conditions were also the two most deactivating (i.e., negative B[93]OLD patterns) conditions in this site (see 3^th^–4^th^ bins filled in blue in Fig 4h) (post-hoc t-test, p-FDR corrected for multiple comparison < 0.05 across bins).

*ROI analysis*. This analysis revealed a significant interaction between language and rendering mode in PMvi (IFG pars opercularis), a region that was selective for the Italian language in late bins (8^th^–9^th^ bins) (Table 1, Figs 3 and 5b, blue). In particular, there was a single peak of higher response to the familiar language with respect to the other three conditions, indicating a clear preferential response to the speech gestures most frequently performed by participants.

By contrast, none of the posterior ROIs (MT+/V5, FFA, pITS, TVSA) nor PMvs/PMd showed an interaction between language and rendering mode.

Finally, we calculated a minimum effect size of 0.15 corresponding to the lowest significant F value reported in Table 1 (F(11,209) = 3.26) [93–95]. A partial eta-squared of 0.15 indicates a large effect [93].

## Discussion

We reported differential brain responses to visual speech kinematics, language familiarity and their interaction. Neuroimaging data showed that language familiarity and temporal rendering of silent speech video-clips modulated two distinct areas in the ventral occipito-temporal cortex. Furthermore, language familiarity modulated the left dorsal premotor cortex, while natural familiar language activated the left ventral premotor cortex in the frontal operculum. These results may indicate that phono-articulatory regions resonate in response to the visemes (visual equivalents of phonemes) of a familiar language. Since in our experiments participants generally did not decode the semantic and syntactic content of visual speech, we propose that these results are confined to the visual equivalent of the phonemic axis. Indeed, our results are in agreement with the definition of a phonological pathway more dorsal with respect to the lexical and semantic pathways, which includes IPS, the dorsal premotor region and the pars opercularis of IFG [96,97].

### Sensitivity to the time-arrow of visual speech

Participants were able to discriminate above chance level visual speech gestures rendered forwards from those rendered backwards. Behavioural results were consistent across experiments. Noteworthy, the sensitivity index d’ estimated in the preliminary and in the main fMRI experiments were not statistically different. These results indicate a sensitivity of the central nervous system for temporal features (i.e., time arrow) of the visible speech, in agreement with the results obtained with a familiar language in a previous study with a similar task [1].

Although lip-reading accuracy of hearing people is generally low and idiosyncratic [98], one cannot rule out a priori that observers were occasionally able to lip-read excerpts of Italian texts in the forward mode, and to use these instances as a cue for discriminating the rendering mode. However, the fact that sensitivity was not significantly different for Italian and Arabic stimuli suggests that lexical competence and speech intelligibility did not play a significant role in the task. The evidence suggests instead that better than chance performance was achieved mainly by a kinematic analysis of movements. If so, the performance reflected the ability to discriminate the motor sequences that are visually perceived as plausible from those that are perceived as implausible from the motoric point of view. This assumption can be sharpened by taking into account the response bias, which describes the position, along the decision axis, of the internal threshold for discriminating the stimuli [76]. In our experiment, there was a response bias in favour of the response “Forward” for the Italian but not for Arabic stimuli, indicating a corresponding shift of the threshold to higher values. This invites the inference that in order to classify a movie reversed in time as ‘Backward’, it is necessary to detect more motoric incongruences in Italian than in Arabic stimuli. This inference is in keeping with the suggestion [99,100] that a high threshold for detecting speech kinematic anomalies favours the stability of speech perception in environments where such anomalies in a familiar language occur due to inter-individual differences or phonetic peculiarities typical of particular social environments (regional inflexions, slang, etc.). Indeed, the participants to both the preliminary and fMRI experiments had no reason to suspect that in half of the video-clips the language being spoken was not Italian.

In a follow-up experiment, participants were asked to identify the language (Italian or not Italian) spoken by the actors in the silent movies. If participants benefitted from speech intelligibility, then the sensitivity to discriminate languages should be higher for Forward than that for Backward rendered stimuli [101]. The results of the follow-up experiment do not conform with this scenario, because the sensitivity index d’ was comparable for Forward and Backward rendered video-clips (see Fig 1), making unlikely that speech intelligibility or lexical processes occurred in our tasks.

The lack of significant correlation across participants between the sensitivity in the fMRI and follow-up experiments suggests that different processes, likely taking into account non-overlapping sets of cues, underlie language and rendering mode discrimination. Conversely, the finding that response bias during rendering mode discrimination depended on the language (and vice-versa) might indicate that, during a late stage of analysis, these signals are merged. This merging might take place in the premotor cortex, where the PMvs/PMd selected the familiar language independently of rendering, while the PMvi responded to the Italian language selectively in the natural kinematics condition.

### Ventral occipito-temporal cortex (vOTC)

The main effects of language and rendering mode activated distinct regions in ventral occipito-temporal cortex. In particular, the comparison of Forward with Backward conditions showed a differential pattern of BOLD responses in the left posterior-middle fusiform gyrus (FG_b_), while the posterior fusiform bilaterally (FG_a_), the inferior occipital gyri and the occipito-temporal sulcus (OTS) were differentially involved with Italian versus Arabic video-clips. The fusiform sites are located posteriorly to the visual areas engaged by semantic and/or lexical processes in the vOTC, which are typically reported at y-coordinates < -50 mm, in the anterior part of the fusiform gyrus [see 102]. Conversely, the fusiform face area (FFA), a region responding selectively to static faces, is located in the posterior region of the fusiform gyrus [35]. The stereotaxic coordinates of FFA centre of mass ([34 –62 –15], [89]) roughly correspond to those of the peak of FG_a_ reported here, but are posterior to those we found for the main effect of rendering mode (FG_b_). Previous studies reported that multiple sites in FG respond to faces [32,89,103]. It is likely that different foci in FG encompass distinct functional modules, as suggested by early PET studies [104,105] showing that gender and face identification activated distinct regions in posterior and middle fusiform gyrus, respectively.

It has been shown that the kinematics of biological movements [30,106–108], as well as the temporal unfolding of faces that express an emotional state [31] engage ventral OTC. In particular, observing facial speech gestures activates FG, although it is unclear whether these activations are specific for speech because some control stimuli, such as gurning faces, activated this region more than talking faces [41]. Indeed, the difference between speech and control stimuli may have been due to differences in low-level features, such as visual motion speed [38,41]. In our study, all four experimental conditions (showing exclusively the lower portion of faces) were comparable in terms of low-level features, such as mean luminance and motion speed. By contrast, time reversal of visual speech stimuli violates motor constraints, and hence produces movements with an implausible kinematics, never occurring during real speech. Previous studies showed that coherent sequences of facial expressions engage the posterior fusiform gyrus more than a scrambled sequence [109]. A possibility is that ventral OTC processed specific kinematic cues embedded in visible speech. Therefore, we speculate that the posterior-middle fusiform site (i.e., FG_b_) was sensitive to the kinematic plausibility of speech gestures. Conversely, the more posterior site FG_a_ was involved mainly in processing kinematic features related to the familiarity of speech, such as the rhythm of speech that is invariant under time reversal but differs across languages (see Introduction).

The previous observations challenge one of the most prominent models about face processing, namely the model proposing that static and dynamic face features are processed separately in ventral OTC and STS, respectively [110]. In fact, our data suggest that ventral OTC has foci sensitive to spatio-temporal (i.e. changeable) characteristics of speech lip movements.

Italian-speech stimuli evoked a higher activity peak than Arabic-speech stimuli in the more posterior site of fusiform gyrus (FG_a_), whereas the sustained post-peak activity was greater for Arabic than Italian stimuli. The Arabic-speech stimuli were unfamiliar, and thus they were presumably unexpected. It is thought that a specific class of neurons (the so-called error neurons) responds selectively to unexpected or unusual stimuli [111–113]. These neurons compare the sensory input with an internally generated (prediction) signal coding what is expected in a given context [114]. In case of a mismatch between the predicted and the incoming sensory signal, error units enhance their activity. We surmise that the greater activity for Arabic than Italian stimuli in the post-peak period might be related to the activity of error units. Therefore, depending on the language (familiar or unfamiliar), this class of neurons contributed differently to the overall neural activity in FG_a_, so the pattern of neural activity changes according with language. However, this mechanism is not specific to ventral OTC, but is a widespread mechanism governing several brain processes (see Friston 2010). For instance, we recently surmised that this kind of neural processing occurs also in lateral OTC when observing unfamiliar walking movements [107,108], and it might even bias balance control [115].

### Lingual gyrus

The interaction between language and rendering mode showed significant activations in the lingual gyrus. Previous reports have already suggested that visually presented speech gestures engage this site [36,116]. The novel finding reported here is that LG responds differently depending on the language. In the follow-up experiment (language discrimination), the d’ was similar between Forward and Backward stimuli, while there was a significant bias toward Arabic-speech or Italian-speech response in the Backward or Forward condition, respectively. The temporal profile of LG activity distinguishes the experimental conditions. In particular, the conditions Arabic Backward and Italian Forward were the two most deactivating conditions. Therefore, the response bias in language discrimination task could be related to a decrease of LG activity. Interestingly, in the auditory domain, LG activity has been found to depend on the familiarity of the spoken language [117]. In the latter study, hearing a speech segment in a second language modulated LG activity differently depending on the participant’s proficiency in that language. Because LG is involved, together with fronto-parietal regions, in speech control [118], these findings suggest a supra-modal effect of speech language in LG, probably due to feedback from high-order centres.

### Premotor prefrontal activity reflects motor simulation of speech gestures

The interaction of rendering mode and language showed a significant effect in the left IFG, comprising the pars opercularis (BA 44), which we have labelled as PMvi, and the pars triangularis (BA45). Most authors agree that the Broca’s area includes both BA 44 and BA 45 of the left hemisphere [43,119–121]. Broca’s area was initially thought to be involved only in speech production, but current research shows that it has a more complex role possibly involving also speech comprehension [43,64,122,123].

In particular, the activation of IFG during speech perception has been interpreted by some authors as the occurrence of a motor simulation of the observed movements. This idea is in accordance with the motor theory of language holding that a simulation of speech gestures in the motor regions is instrumental for speech perception and understanding [53,124–126]. However, it is still an open issue whether the IFG activation during speech perception is related to a language-specific process, as the putative motor simulation of speech gestures, or represents a general-domain cognitive mechanism [127]. It has also been suggested that distinct IFG foci have different roles during speech perception. Specifically, articulatory rehearsal of speech gestures would occur in pars opercularis, while pars triangularis and orbitalis could be related to cognitive-control mechanisms, such as decisional or working-memory processes [68,128,129]. The rehearsal function of pars opercularis generalizes across different types of movement, as this region was found to respond also to observation of hand movements [130].

The time-course of the BOLD signal that we observed in the pars opercularis of IFG fit with the predictions of the motor simulation hypothesis (Figs 3 and 5b). According to this hypothesis, the familiar stimuli (i.e., Italian Forward) should elicit a higher level of activity than unfamiliar, implausible gestures difficult to reproduce (e.g., Italian Backward or Arabic Forward and Backward stimuli). Conversely, in the pars triangularis of IFG, Italian-speech stimuli and Arabic-speech stimuli, for which latter participants had no motoric expertise, evoked comparable responses although shifted in time (Figs 3 and 4g). Thus, the results do not suggest a specific sensitivity for Italian-speech stimuli in the pars triangularis of IFG, but rather sensitivity for the kinematics of natural mouth movements, a kind of biological motion. Our data, limited to the case of a familiar language (Italian), are also in agreement with those reported by Paulesu et al. [27] in which IFG activity was greater for forward than backward silent movies of a speech in a familiar language.

The issue of the role of intelligibility of silent visual speech should be further investigated, as one could argue that motor simulations occur only or mainly when linguistic competences are required, as with a lexical discrimination task [27,86,131]. However, we believe that the ability of participants to speech-read might be a confound when trying to disentangle motor from higher cognitive functions of Broca’s area. In our case, it appears that motor simulation occurs in absence of comprehension of the content of the speech.

### Summary and conclusions

Previous studies focused mainly on the role of temporal auditory regions [37,48,132] and frontal regions [86,131,133] in processing visual speech. More recently, it has been shown that a region in the left posterior temporal cortex, the so-called temporal visual speech area (TVSA), is activated in visual phonetic discrimination [38], possibly integrating information coming from high-level visual areas in OTC [3,41,134]. We did not find significant effects in TSVA, as verified through a specific ROI drawn in this region. Our data suggest that the ventral occipito-temporal cortex has a sensitivity to visual speech gestures, contrary to the view that the peculiar analysis of visual speech starts at higher cortical levels [27,135]. Our results support the hypothesis that kinematic cues embedded in visible speech can be extracted through the visual pathways [136], outside the classical areas related to auditory speech and audio-visual integration [36,37,116,137,138]. Finally, the selective responses of PMvs / PMd to the familiar language and of PMvi to the natural familiar language support the hypothesis that motor simulation drives premotor activity during visible speech perception.

## Supporting information

S1 Video (AVI)

S2 Video (AVI)

S3 Video (AVI)

S4 Video (AVI)
